# Early removal of post-operative scabs after follicular unit extraction using a 2% salicylic acid keratolytic shampoo: a multicentre descriptive pilot study

**DOI:** 10.3389/fmed.2026.1741064

**Published:** 2026-05-01

**Authors:** Teresa Meyer, Pablo Fernández-González, Alia Shafiq-Sánchez, Alba Gómez-Zubiaur

**Affiliations:** 1 Meyer & Alcaide, Centro Sanitario de la Piel y el Pelo, Málaga, Spain; 2 Medical Affairs Department, Cantabria Labs, Madrid, Spain; 3 Trichology Unit, Instituto Médico Ricart, Madrid & Valencia, Spain

**Keywords:** hair transplantation, keratolytic therapy, post-operative scabs, salicylic acid shampoo, scalp wound care

## Abstract

**Background:**

Hair transplantation is increasingly performed for advanced androgenetic alopecia (AGA), yet postoperative scalp care remains poorly standardized. Persistent scabs after follicular unit extraction (FUE) may delay healing, compromise graft survival, and increase the risk of infection.

**Objective:**

To evaluate the effectiveness and tolerability of a keratolytic shampoo containing 2% salicylic acid, piroctone olamine, and a micro-depositing conditioning polymer for the early management of postoperative scabs after FUE.

**Methods:**

A prospective, multicenter, single-arm pilot study was conducted in 21 male patients following a structured 10-day washing protocol starting 48–72 h post-surgery. Investigator-assessed erythema, desquamation, and scab scores were recorded, along with patient-reported symptoms and satisfaction.

**Results:**

Mean scab severity decreased from 2.67 ± 0.97 to 1.76 ± 0.62, indicating effective crust detachment without barrier disruption. Erythema increased transiently post-wash (1.90 ± 0.62 to 2.81 ± 1.21) but was not associated with burning, itching, or pain. The overall mean satisfaction score was 4.05 ± 0.77, with 76.2% of patients agreeing that the shampoo reduced scabs and 67% stating they would recommend it. No adverse events were recorded.

**Conclusion:**

Continuous use of this 2% salicylic acid-based keratolytic cleanser from 48–72 h after FUE promoted gentle scab removal, excellent tolerability, and high patient satisfaction. Incorporating such formulations into early postoperative care may improve outcomes and comfort after hair transplantation.

## Introduction

Hair transplantation is increasingly common in clinical practice and is the treatment of choice for advanced androgenetic alopecia (AGA) when medical therapy cannot restore sufficient density. Indications should be carefully assessed, ensuring disease stability in younger patients, while in older individuals it can be considered as a standalone option (1). The procedure consists of harvesting follicular units that are resistant to hormonal miniaturization and implanting them into bald areas of the scalp. This process inevitably disrupts the epidermis, triggering exudation, fibrin deposition and the development of adherent scales and crusts. Persistent scabs impair effective cleansing, delay re-epithelialization and compromise patient comfort; consequently, immediate post-operative shampooing is generally advised (2). However, standardized protocols for peri-operative care are lacking, and the timing, choice of products, and overall management often rely on individual clinical practice.

In this study we evaluate, under a structured regimen, a keratolytic cleanser containing 2 % salicylic acid designed to solubilize keratin and soften scabs, enabling gentle removal without mechanical trauma (3). The formulation also incorporates piroctone olamine, a broad-spectrum antifungal and antibacterial agent that helps maintain a low bioburden while graft sites remain vulnerable (4), and a micro-depositing conditioning polymer that improves hair shaft lubricity and soothes the inflamed scalp, reducing friction during repeated washes (5). Together these components offer a rational, multifaceted approach to early crust management after follicular unit extraction (FUE).

## Materials and methods

### Study design

A prospective, multicentre, single-arm descriptive pilot study was performed to evaluate the usefulness of a 2% salicylic-acid keratolytic shampoo for the early removal of scabs after FUE.

### Participants

Male patients (age ≥ 18 years) who underwent FUE with complete scalp shaving at two independent hair-restoration clinics in Spain were consecutively enrolled. Inclusion criteria were: (i) willingness to comply with study visits, and (ii) absence of known hypersensitivity to any shampoo component. Exclusion criteria comprised: (i) concomitant topical therapies on the recipient area, (ii) dermatological disorders that could interfere with wound healing, and (iii) systemic conditions or medications known to impair skin repair. All participants provided written informed consent.

### Intervention

Starting 48–72 h post-surgery, patients washed the recipient area with the investigational shampoo twice daily for 10 days, the protocol is summarized in Table 1. To mitigate the risk of bias, assessments were conducted using predefined, standardized scales under a uniform multicenter protocol, and all outcomes were prospectively collected and transparently reported.

**Table 1:** Overview of postoperative washing protocol and data collection schedule.

Post-operative day	Procedure in clinic	Home instructions	Data collected
Day 0	Hair-transplant procedure (FUE)	—	—
Day +2 or +3	First supervised wash immediately after surgery; patients were shown step-by-step cleansing technique.	—	No formal scoring; only photographic documentation of baseline crusts.
Days +3 to +7	—	Gentle shampooing twice daily using fingertip massage and low-pressure rinse.	None (self-care period).
Days +7 to +10	Second clinic visit. A vigorous wash is performed to detach scabs. Pre-wash and immediate post-wash evaluations are carried out.	Continue twice-daily washes at home with gradually increased pressure until scabs disappear.	• Investigator 5-point scales (erythema, scaling, scabs) – pre/post. • VAS for itch & pain – pre/post. • Macro & trichoscopic photographs – pre/post. • 13-item patient questionnaire (single administration, post-wash) (Table 2). • 5-item physician usability survey (post-wash).

The table summarizes in-clinic procedures, home care instructions, and assessments performed at each study timepoint following follicular unit extraction. VAS, Visual Analog Scale; Investigator 5-point scale (1 = none, 2 = mild, 3 = moderate, 4 = severe, and 5 = very severe).

**Table 2:** Post-wash patient questionnaire assessing product usability and subjective satisfaction rated on a 5-point Likert scale (1 = strongly disagree, 2 = disagree, 3 = neutral, 4 = agree, 5 = strongly agree).

#	Questionnaire Statement
1	The shampoo has a pleasant texture.
2	The shampoo's fragrance is pleasant.
3	It is easy to apply the shampoo to the scalp and it spreads well along the hair shaft.
4	It is easy to distribute and massage the shampoo over the scalp.
5	The shampoo rinses out easily without leaving residue.
6	I feel that my scalp stays cleaner after using the shampoo and for a longer time.
7	Using shampoo has reduced the amount of scabs on my scalp.
8	I have noticed a reduction in scalp irritation since I started using the shampoo.
9	I am satisfied with the shampoo's effectiveness in removing scales.
10	The shampoo has improved the overall appearance of my scalp.
11	My hair remains clean for a longer period.
12	The shampoo leaves a satisfactory finish and shine on my hair.
13	I would recommend this shampoo to other patients who have undergone a hair transplant.

### Product of study

The shampoo contained 2% salicylic acid (keratolytic), piroctone olamine (antifungal), and a conditioning polymer (Plant-derived micro-spheres) to minimize irritation. No other cleansing or topical products were allowed during the study period.

### Image acquisition

Macro-level and trichoscopic photographs were obtained at each visit to document treatment progress. Standardized distance and lighting were used for the macro images, while trichoscopy was performed at × 20 and × 40 magnification with a digital capture device.

### Statistical analysis

Given the pilot nature of the study and the small sample size (*n* = 21), data were summarized using descriptive statistics. Continuous variables were expressed as mean ± standard deviation (SD) or median (interquartile range [IQR]), as appropriate, and categorical variables as frequencies and percentages. In line with the exploratory design, no inferential statistical analyses were planned or performed.

### Ethical considerations

The study was conducted in accordance with the ethical principles of the Declaration of Helsinki and relevant local regulations. Because the project involved a descriptive evaluation of routine post-operative care without any additional intervention beyond usual clinical practice, formal approval by an institutional review board was not required under national guidelines. All participants received written and verbal information about the purpose, procedures, and data handling of the study, and each provided written informed consent for participation and for the publication of anonymized clinical images and results. All data were coded to ensure patient confidentiality, and no identifying information is presented in this manuscript.

## Results

Twenty-one male patients (mean ± SD age = 44 ± 10 years; range 26–60) completed the protocol.

### Investigator-rated outcomes and patient-reported VAS (Day +7/+10)

**Figure 1:**
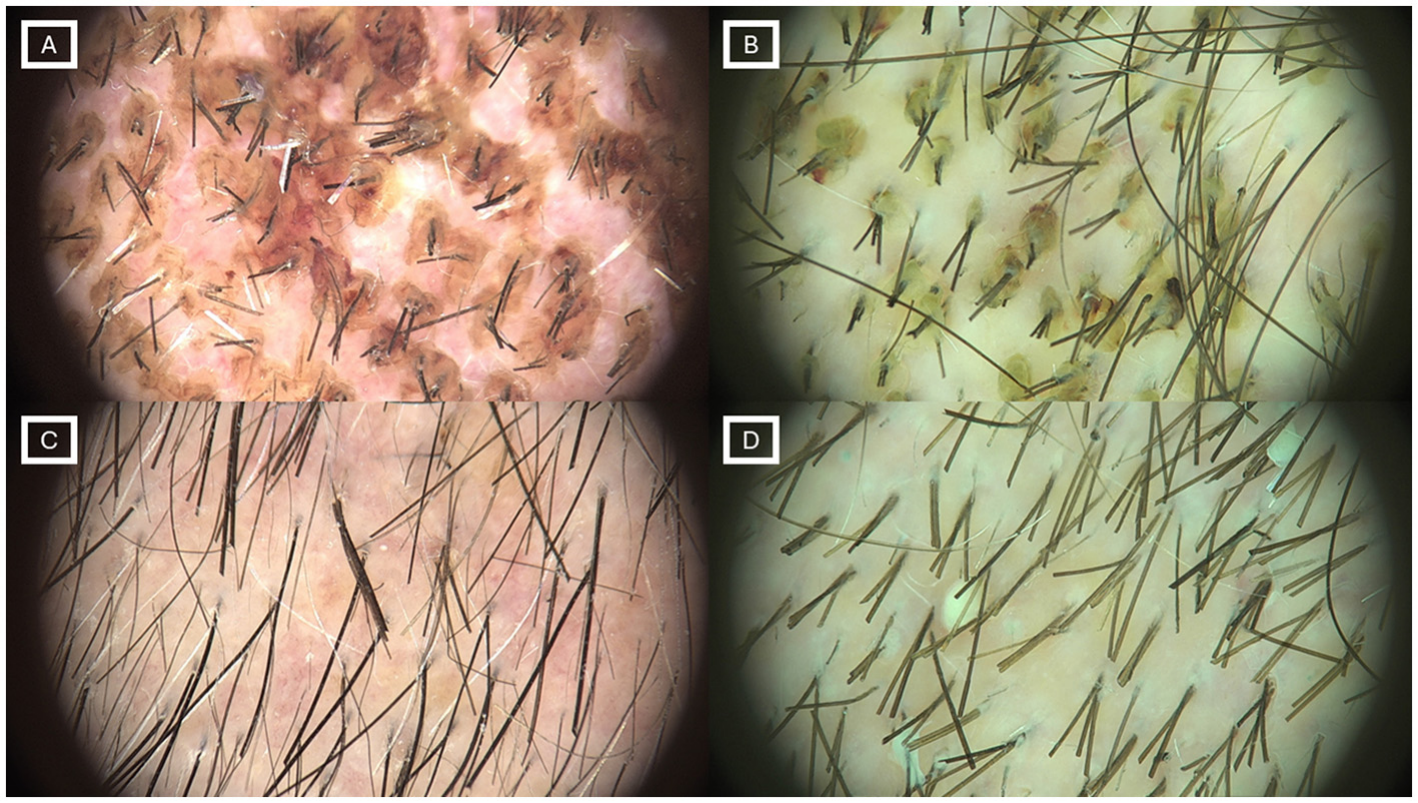
Trichoscopic images illustrating postoperative scalp evolution, representing clinical examples selected to illustrate typical findings observed during follow-up. Panels (**A)** and (**B)** correspond to two different volunteers at the second visit, immediately before the first supervised wash. Typical perifollicular adherent scabs can be observed, a characteristic finding during the early postoperative period. Panels (**C)** and (**D)** show the same volunteers at the third visit, immediately before the final wash at the clinic, demonstrating a marked reduction in perifollicular crusting and overall improvement in scalp appearance.

Scab severity decreased from a “moderate” mean of 2.67 ± 0.97 (range 1–5) at V1 to 1.76 ± 0.62 (range 1–3) at V2, indicating a shift towards the “absent/mild” category in most participants (Figure 1).

Baseline desquamation was low, with a mean of 1.52 ± 0.60 (range 1–3); the value remained virtually unchanged post-wash (1.52 ± 0.60, range 1–3).

At the pre-wash assessment (V1) mean erythema was 1.90 ± 0.62 (range 1–3). Immediately after the supervised wash (V2) it rose to 2.81 ± 1.21 (range 1–4).

Patient-reported symptoms after the wash were modest: itch scored 2.05 ± 1.53 on the VAS (range 0–5) and pain 0.52 ± 0.81 (range 0–2) at the post-wash evaluation.

### Patient-reported usability and satisfaction

The 13-item Likert questionnaire at the end of the study confirmed a high level of acceptance (Table 3). The overall mean score across all items was 4.05 ± 0.77 (1 = strongly disagree, 5 = strongly agree), with individual item means ranging from 3.5 to 4.7.

**Table 3:** Patient-reported usability and satisfaction outcomes (*n* = 21).

Questionnaire statement	Mean + (SD)	% Top-2-Box (4-5)
The shampoo has a pleasant texture.	4.62 (0.49)	100%
The shampoo's fragrance is pleasant.	4.10 (0.83)	81%
It is easy to apply the shampoo to the scalp and it spreads well along the hair shaft.	4.48 (0.6)	95.2%
It is easy to distribute and massage the shampoo over the scalp.	4.67 (0.48)	100%
The shampoo rinses out easily without leaving residue.	4.29 (0.71)	85.7%
I feel that my scalp stays cleaner after using the shampoo and for a longer time.	3.62 (0.74)	47.6%
Using shampoo has reduced the amount of scabs on my scalp.	4.15 (0.87)	76.2%
I have noticed a reduction in scalp irritation since I started using the shampoo.	4.00 (0.77)	71.4%
I am satisfied with the shampoo's effectiveness in removing scales.	3.86 (1.1)	57.1%
The shampoo has improved the overall appearance of my scalp.	3.48 (0.87)	42.9%
My hair remains clean for a longer period.	3.52 (0.81)	42.9%
The shampoo leaves a satisfactory finish and shine on my hair.	3.71 (0.84)	66.7%
I would recommend this shampoo to other patients who have undergone a hair transplant.	3.95 (0.92)	66.7%

Results of the 13-item patient questionnaire assessing product acceptability and perceived effectiveness at the end of the study period. Responses were rated on a 5-point Likert scale (1 = strongly disagree; 2 = disagree; 3 = neutral; 4 = agree; 5 = strongly agree). Values are presented as mean scores and percentage of top-2-box responses (scores 4–5). SD, standard deviation.

No adverse events related to the shampoo were recorded during the study, and none of the twenty-one participants withdrew for safety or tolerability reasons.

## Discussion

For patients with moderate to advanced stages of AGA, hair transplantation represents one of the preferred interventions to restore hair density and improve scalp coverage, often complementing ongoing medical therapies aimed at stabilizing disease progression (1, 2, 6).

Preoperative assessment and surgical techniques in hair transplantation have become progressively standardized, improving safety, graft planning, and overall clinical outcomes. As the procedural aspects of FUE are now highly protocolized, increasing attention has shifted toward optimizing postoperative management, particularly early scalp care and scab removal (7). FUE has progressively become the dominant method due to its minimally invasive nature, faster recovery, and favorable cosmetic profile, although follicular unit transplantation (FUT) remains valuable for high-yield cases (6).

Within postoperative care, one of the aspects most strongly emphasized in the consensus recommendations is the washing protocol for both the donor and recipient areas immediately after the procedure (8).

In fact, particular emphasis is placed on the importance of proper scab removal, as difficult to remove scabs should be managed in a clinical setting to prevent graft loss or postoperative infection (9–11). This can be achieved using a gentle, non-irritating shampoo or by applying emollient oils to soften the scabs (7, 11). Clinically, 0.75% hydrogen peroxide or 0.9% saline may also be used to facilitate softening and removal of persistent scabs.

In our study, the shampoo was very well accepted by the volunteers, who reported a high subjective perception of effective scab removal. This aligns with the well-established keratolytic activity of 2% salicylic acid (3, 4). The shampoo formulation not only allows the active ingredient to act directly on postoperative scabs but also enhances its efficacy through the combined cleansing action of the vehicle and the mechanical effect of washing and rubbing. Interestingly, post-wash assessments consistently showed a transient increase in erythema, which was not accompanied by sensations of intense burning, itching, or pain, suggesting that the procedure was well tolerated and did not induce clinically relevant irritation. This might be explained by the heightened vasoreactivity of the scalp in the early postoperative phase, which makes it more responsive to even minimal external stimuli (11). Gentle mechanical friction during shampoo application and rinsing can transiently increase superficial blood flow, leading to a visible but short-lived erythema. This vasodilatory response likely reflects a physiological vascular reaction rather than pathological inflammation, which is consistent with the absence of accompanying symptoms.

Desquamation scores remained low and stable throughout the shampoo intervention period, with no measurable change immediately after washing. This likely reflects the limited baseline presence of scaling in the postoperative setting, where scab formation predominates over diffuse desquamation. Moreover, the keratolytic action of 2% salicylic acid appears to have effectively targeted adherent scabs without significantly altering the underlying stratum corneum physiology (3), which may explain the absence of additional flaking or barrier disruption. This stability suggests that the formulation exerted its intended action, supporting its favorable tolerability profile.

Regarding other subjective items collected, the product received excellent feedback from participants. Texture, application, and rinsability were particularly well rated, and 71% of subjects selected the top two categories (“excellent” or “very good”) for irritation and scalp comfort, as well as for scab reduction and general product acceptability. This positive perception is clinically relevant in the context of post-transplant care, where patients require gentle, non-irritating products that minimize scalp disturbance during the early healing phase (7, 8). Moreover, the formulation demonstrated excellent tolerability and was not associated with any adverse effects, further supporting its suitability for postoperative scalp care.

This study has limitations inherent to its single-arm, non-controlled pilot design, which precludes establishing a causal relationship between the intervention and the observed improvements. As scab resolution is part of natural postoperative healing after FUE, some benefit may reflect physiological recovery rather than the shampoo itself. The small sample size further limits generalizability and precludes definitive conclusions. Larger randomized controlled trials including more diverse populations and appropriate comparators are therefore required to confirm these findings and more precisely isolate the specific effect of the keratolytic regimen.

## Conclusion

Hair transplantation has become an important therapeutic option for patients with advanced forms of AGA, with the FUE technique currently being the most widely adopted procedure. Postoperative care plays a crucial role in preventing complications such as infection and in promoting graft survival. Among the most frequent concerns during the postoperative period is the management and removal of scabs that form on the scalp following transplantation, as their persistence can compromise the aesthetic outcome and delay healing. In this context, the use of a shampoo formulated with 2% salicylic acid, piroctone olamine and a micro-depositing conditioning polymer appeared to facilitate gentle removal of postoperative scabs when applied from 48–72 h after surgery until approximately day 10. The product demonstrated good cosmetic acceptability and tolerability, with no relevant adverse effects reported. However, given the exploratory pilot design, these findings should be considered preliminary and hypothesis-generating and require confirmation in controlled studies before definitive conclusions regarding efficacy can be drawn.

## Data Availability

The raw data supporting the conclusions of this article will be made available by the authors, without undue reservation.

## References

[1] Vañó-Galván S Fernandez-Crehuet P Garnacho G Gómez-Zubiaur A Hermosa-Gelbard A Moreno-Arrones OM . Recommendations on the clinical management of androgenetic alopecia: a consensus statement from the spanish hair disorders group of the spanish academy of dermatology and venererology (AEDV). Actas Dermosifiliogr. (2024) 115:T347–55. doi: 10.1016/j.ad.2023.10.043 38336246

[2] Jimenez F Vogel JE Avram M CME. article Part II. Hair transplantation: surgical technique. J Am Acad Dermatol. (2021) 85:818–29. doi: 10.1016/j.jaad.2021.04.063 33915242

[3] Bashir SJ Dreher F Chew AL Zhai H Levin C Stern R . Cutaneous bioassay of salicylic acid as a keratolytic. Int J Pharm. (2005) 292:187–94. doi: 10.1016/j.ijpharm.2004.11.032 15725565

[4] Lodén M Wessman C. The antidandruff efficacy of a shampoo containing piroctone olamine and salicylic acid in comparison to that of a zinc pyrithione shampoo. Int J Cosmet Sci. (2000) 22:285–9. doi: 10.1046/j.1467-2494.2000.00024.x 18503415

[5] Dias MFRG. Hair cosmetics: an overview. Int J Trichology. (2015) 7:2–15 doi: 10.4103/0974-7753.153450 PMC4387693 25878443

[6] Rassman WR Bernstein RM McClellan R Jones R Worton E Uyttendaele H. Follicular unit extraction: minimally invasive surgery for hair transplantation. Dermatol Surg. (2002) 28:720–8. doi: 10.1046/j.1524-4725.2002.01320.x 12174065

[7] Vañó-Galván S Bisanga CN Bouhanna P Farjo B Gambino V Meyer-González T . An international expert consensus statement focusing on pre and post hair transplantation care. J Dermatolog Treat. (2023) 34:2232065. doi: 10.1080/09546634.2023.2232065 37477225

[8] Shichang L Jufang Z Xiangying Y Yali W Li N. Self-management in the post-hair transplantation recovery period among patients with androgenetic alopecia: a qualitative study. Int J Nurs Stud Adv. (2024) 7:100234. doi: 10.1016/j.ijnsa.2024.100234 39282021 PMC11401157

[9] Kerure AS Patwardhan N. Complications in hair transplantation. J Cutan Aesthet Surg. (2018) 11:1829. doi: 10.4103/JCAS.JCAS12518 PMC6371733 30886471

[10] Khatib M Skorochod R Wolf Y. Complications following hair transplantation: a systematic literature review and meta-analysis. Aesthetic plast surg. (2025) 49:6393–405. doi: 10.1007/s00266-025-05125-y 40913181

[11] Huang J Peng Y Zhou W Chen D Guo L Guo J. Summary of best evidence for perioperative management practices in hair transplantation patients : facial skeleton. Aesthetic Plast Surg. (2024) 48:4791–804. doi: 10.1007/s00266-024-04360-z 39377788

